# Investigation of Far Infrared Emission and UV Protection Properties of Polypropylene Composites Embedded with Candlenut-Derived Biochar for Health Textiles

**DOI:** 10.3390/molecules29204798

**Published:** 2024-10-10

**Authors:** Rayland Jun Yan Low, Pengfei He, Junianto, Ningyu Qiu, Amanda Jiamin Ong, Hong Han Choo, Yosia Gopas Oetama Manik, Rikson Siburian, Ronn Goei, Stephen F. Burns, Alfred Iing Yoong Tok, Vitali Lipik, Boon Peng Chang

**Affiliations:** 1 School of Materials Science and Engineering, Nanyang Technological University, 50 Nanyang Avenue, Singapore 639798, Singapore; rayland.low@hotmail.com (R.J.Y.L.); pengf.he@gmail.com (P.H.); junianto.sp.15@gmail.com (J.); qiuningyu@gmail.com (N.Q.); jiamin.ong@ntu.edu.sg (A.J.O.); honghan001@e.ntu.edu.sg (H.H.C.); ronn.goei@gmail.com (R.G.); miytok@ntu.edu.sg (A.I.Y.T.); 2 Department of Chemistry, Faculty of Mathematics and Natural Sciences, Universitas Sumatera Utara, Medan 20155, Indonesia; yosiagopas01@gmail.com (Y.G.O.M.); rikson@usu.ac.id (R.S.); 3 Physical Education and Sports Science, National Institute of Education, Nanyang Technological University, 1 Nanyang Walk, Singapore 637616, Singapore; stephen.burns@nie.edu.sg

**Keywords:** far infrared radiation, polymer-matrix composites (PMCs), optical properties/techniques, thermal properties, candlenut-derived biochar

## Abstract

Far infrared radiation (FIR) within the wavelength range of 4–14 μm can offer human health benefits, such as improving blood flow. Therefore, additives that emit far infrared radiation have the potential to be incorporated into polymer/fabric matrices to develop textiles that could promote health. In this study, biochar derived from candlenuts and pyrolyzed with activated carbon (AC) was incorporated into polypropylene (PP) films and investigated for its potential as a health-promoting textile additive. The properties of biochar were compared with other far infrared (FIR) emitting additives such as hematite, Indian red ochre, and graphene. The addition of biochar increased FIR emissivity to 0.90, which is 9% higher than that of pristine PP. Additionally, biochar enhanced UV and near-infrared (NIR) blocking capabilities, achieving an ultra-protection factor (UPF) of 91.41 and NIR shielding of 95.85%. Incorporating 2 wt% biochar resulted in a 3.3-fold higher temperature increase compared to pristine PP after 30 s of exposure to an FIR source, demonstrating improved heat retention. Furthermore, the ability to achieve the lowest thermal effusivity among other additives supports the potential use of biochar-incorporated fabric as a warming material in cold climates. The tensile properties of PP films with biochar were superior to those with other additives, potentially contributing to a longer product lifespan. Additionally, samples with red ochre exhibited the highest FIR emissivity, while samples with hematite showed the highest capacity for UV shielding.

## 1. Introduction

Far infrared (FIR) radiation falls within the electromagnetic spectrum range of 750 to 1000 μm. Objects above absolute zero temperature, such as the human body at room temperature, emit FIR. This radiation is perceived by human thermoreceptors as radiant heat. Due to the human body’s high water content, it can effectively absorb FIR, causing the molecules within the body to undergo various vibrational motions [1]. FIR radiation within the range of 4–14 μm, also known as “life rays”, can penetrate the human skin and induce heat production through resonance effects via intramolecular micro-vibrations [2]. These resonance processes can promote cellular functions by dilating capillaries and enhancing blood circulation [3].

Furthermore, studies have shown that FIR can enhance the viability of cells and tissues, decrease the sensitivity of neuroblastoma cells to rotenone (an inhibitor of mitochondrial respiration), and alleviate mitochondrial fragmentation. This, in turn, helps prevent the onset of Spinocerebellar ataxia type 3, which is associated with neurodegenerative diseases [4].

In recent years, the health benefits of applying FIR have been explored through human trials utilizing fabrics incorporated with far infrared-emitting additives. For instance, bedsheets integrated with far infrared-emitting bioceramics were found to alleviate insomnia symptoms and reduce daytime napping in a randomized controlled trial involving 29 adults. This effect may be attributed to nitric oxide (NO)-induced vasodilation, potentially increasing perfusion and skin temperature [5]. Another study involving 12 physically active male cyclists demonstrated a decrease in oxygen consumption during low-intensity exercise when participants wore clothing containing far infrared-emitting ceramics, as opposed to wearing standard apparel. It was suggested that the ceramics facilitated the enhanced release of NO, resulting in vasodilation and improved oxygen supply to muscles during low-intensity cycling [6]. Infrared radiation can also result from changes in dipole moment due to molecular vibrations [7]. For instance, tourmaline exhibits high FIR emissivity, even at low temperatures, owing to its atomic arrangement. However, a slight temperature change can modify the dipole moment of molecules within the material, thereby enhancing the release of infrared radiation due to heightened molecular excitation [8]. Previous studies [9] also indicated that iron oxide-based natural mineral additives possess superior FIR emissivity, likely due to molecular vibrations at FIR wavelengths. According to Kirchoff’s law of emission, absorption at FIR wavelengths equals emission in thermal equilibrium at the same wavelength.

In addition to inorganic fillers, carbon nanostructures like graphene have also shown promising infrared emissivity attributed to the presence of π electron optical transitions [10]. Zhao et al. [11] synthesized graphene/MnO_2_ composites via a hydrothermal method, which reportedly exhibited higher emissivity than pristine MnO_2_. The rationale behind this enhancement in FIR emissivity lies in the specific large area resulting from an increased molecule count on the exposed surface, consequently elevating surface energy. Apart from enhanced FIR emissivity, graphene and natural mineral additives like hematite also possess other functional properties such as UV protection [12,13] and excellent thermal conductivity [14,15].

Despite possessing these functional properties, both hematite and graphene exhibit low compatibility in forming a strong polymer/filler interface with non-polar polymers such as polypropylene. Although it’s anticipated that all functional groups are eliminated when graphene oxide is reduced to graphene, achieving 100% pure graphene remains challenging, often resulting in the presence of polar functional groups like carboxylic acid. Additionally, obtaining high-purity graphene is costly, and concentrations exceeding 0.5 wt% should be used judiciously, particularly when not incorporated into smart textiles that leverage its superior electrical properties. Hematite, being a polar compound, has been shown to enhance mechanical and wettability properties when added to polar polymers such as polyurethane [16].

To enhance the compatibility of polar additives with non-polar polymers, various methods have been proposed, including the addition of compatibilizers such as maleic anhydride grafted polymers [17], and the utilization of coupling agents such as silanes [18] to promote surface compatibility, as well as in situ polymerization [19]. However, these techniques come with drawbacks, including increased costs. In situ polymerization is limited to low-viscosity polymers to ensure effective reactions [20]. Moreover, optimizing the amount of coupling agents and compatibilizers is crucial, as excessive use of these chemicals may lead to a plasticizing effect, reducing stiffness, hardness [21], and strength [22]. Hence, there is a demand for additives containing non-polar groups or those that can be chemically modified using simple methods without the need for expensive chemicals.

Biochar has garnered increasing attention over the years since its production from the pyrolysis of biowaste, promoting the utilization of waste to support sustainability [23,24]. Moreover, its low density renders it suitable for lightweight polymer applications, particularly in textile fabrication [25]. One of the most attractive features of biochar is its tunable functionality based on pyrolysis temperature. A study conducted by Watt et al. [26] investigated the effect of pyrolysis temperature on polymer-filler interactions by adding biochar obtained at different temperatures to polyamide. The results revealed that when biochar was pyrolyzed at temperatures below 600 °C, polar functional groups such as -OH were abundant, facilitating a strong polymer-filler interface with polar polymers like polyamide, thus improving mechanical properties. Conversely, when pyrolyzed at temperatures above 600 °C, the abundance of polar groups in the biochar decreased due to deoxygenation and dehydration. This suggests that such biochar can form a better polymer-filler interface with non-polar polymers like polypropylene (PP) [27]. Despite the tunable functional groups of biochar, achieving a lower abundance of polar functional groups to ensure compatibility with non-polar polymers such as PP requires high temperatures. Therefore, there is interest in obtaining biochar with fewer functional groups at lower pyrolysis temperatures and investigating the FIR emissivity and thermal effusivity of polymer-biochar composites, a task not previously undertaken. While the UV protection properties of biochar-based coatings on stainless steel have been studied [28], their UV protection capabilities for textile applications have not been explored. Hence, this study utilized activated carbon (AC) as an absorbent catalyst in the pyrolysis of candlenut-derived biochar, aiming to facilitate the removal of polar functional groups even at lower pyrolysis temperatures. The resulting pyrolyzed biochar was then mixed with PP through melt blending and subsequently hot-pressed into films to examine the effects of biochar addition on FIR emissivity, UV protection, and thermal effusivity. PP films embedded with graphene, as well as iron-based minerals such as hematite and Indian red ochre, were also studied for comparison.

The study demonstrates that adding biochar can significantly increase FIR emissivity, enhance UV and NIR blocking capabilities, improve heat retention, and maintain superior tensile properties compared to alternative additives. The significance of this study lies in advancing the development of IR-emitting materials for potential future applications in textiles, offering potential health benefits and improved thermal comfort.

## 2. Results and Discussion

### 2.1. Raman and FTIR Spectra of Graphene and Biochar Composites

**Figure 1:**
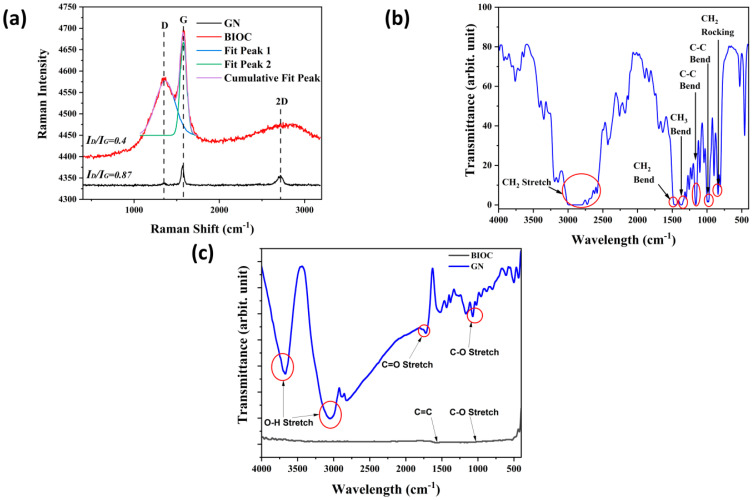
(**a**) Raman Spectra of GN and BIOC from 400 ${cm}^{-1}$ to 3000 ${cm}^{-1}$; (**b**) FTIR Spectra of Neat PP from 4000 ${cm}^{-1}$–400 ${cm}^{-1}$; (**c**) FTIR Spectra of GN and BIOC from 4000 ${cm}^{-1}$–400 ${cm}^{-1}$.

Raman spectroscopy was conducted on both BIOC and GN to analyze the presence of defects and graphitization in carbon-based materials. As depicted in Figure 1a, the peak at 1330 cm^−1^ for both materials was attributed to the D band, indicating the presence of defects in the crystal lattice that disrupt the hexagonal carbon structure [29,30]. The peak at around 1600 cm^−1^ was assigned to the G band, indicating highly ordered graphitic carbons [31]. Additionally, the peak around 2697 cm^−1^ was associated with the 2D band, used to differentiate the number of layers in the carbon structure. A sharp peak was observed for GN, representing a monolayer structure typical of graphene. Conversely, the 2D peak for BIOC was broad, indicating a multi-layered carbon structure [32].

The ratio of the intensity of the D band to the G band, represented as $I_{D}/I_{G}$, is utilized to determine the degree of graphitization in the carbon material. A lower $I_{D}/I_{G}$ ratio signifies a higher degree of graphitization. As shown in Figure 1a, the $I_{D}/I_{G}$ ratio for GN is low, suggesting a very high degree of graphitization and a lower number of defects. However, the $I_{D}/I_{G}$ ratio of 0.87 for BIOC is lower than that reported for biochar from other sources, indicating that composites with BIOC may possess improved mechanical properties [33,34].

FTIR was used to identify the functional groups present in the carbon-based powders and blank PP film to assess their polarity. The FTIR spectra of the pristine PP film was shown in Figure 1b. The peak at 2896 ${cm}^{-1}$ corresponds to the CH_2_ asymmetric stretch. The peaks at 2724 ${cm}^{-1}$, 2619 ${cm}^{-1}$, and 2582 ${cm}^{-1}$ correspond with the CH_2_ symmetric stretch. The peak at 1455 ${cm}^{-1}$ corresponds to the CH_2_ symmetric bend. The peak at 1370 ${cm}^{-1}$ corresponds to the symmetric bending vibration mode of CH_3_ group [35]. The peak at 1161 ${cm}^{-1}$ corresponds to the C-C bending, which is the backbone of polypropylene [36]. The peak at 994 ${cm}^{-1}$ corresponds to the CH_3_ asymmetrical rocking. The peak at 841 ${cm}^{-1}$ corresponds to the CH_2_ rocking vibration [37]. These results are consistent with the chemical structure of PP which only contains hydrocarbon and is non-polar. Figure 1c shows the IR spectra of GN and BIOC powders. In the spectra of GN, the peak at 3670 ${cm}^{-1}$ and 3050 ${cm}^{-1}$ corresponds to O–H group stretching vibrations. The peak in 1722 ${cm}^{-1}$ is assigned to C=O stretching of carboxylic acid. The peak at 1022 ${cm}^{-1}$ is assigned to C–O stretching vibrations [38]. In the spectra of BIOC powders, The peak at 1569 ${cm}^{-1}$ is related to the presence of C=C bonds with conjugation of π electrons and the presence of cyclic alkene [39]. The peak at 1156 ${cm}^{-1}$ is due to the stretching vibration of C–O in alcohol [40].

### 2.2. Far Infrared Emissivity of PP Films with Functional Additives

**Figure 2:**
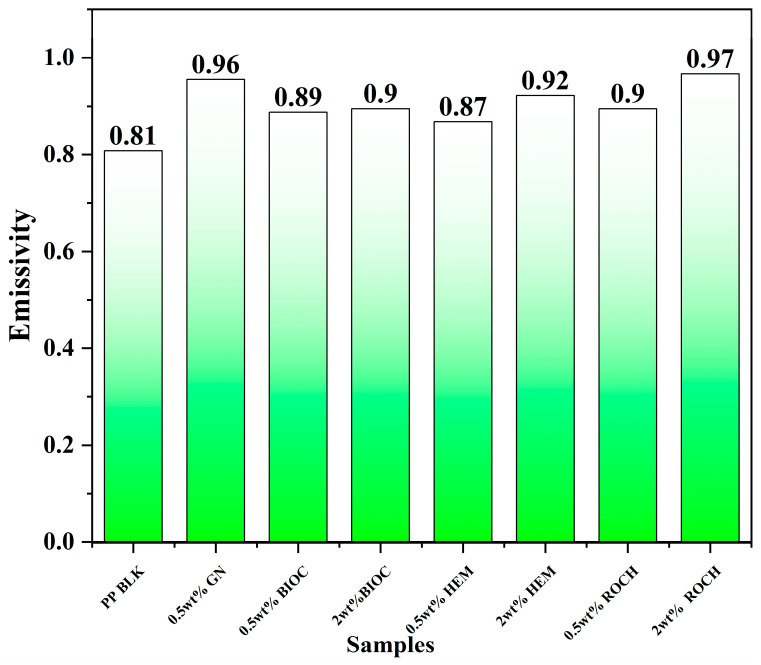
FIR emissivity at 5–14 µm of the PP films with 0.5% and 2% additives.

**Figure 3:**
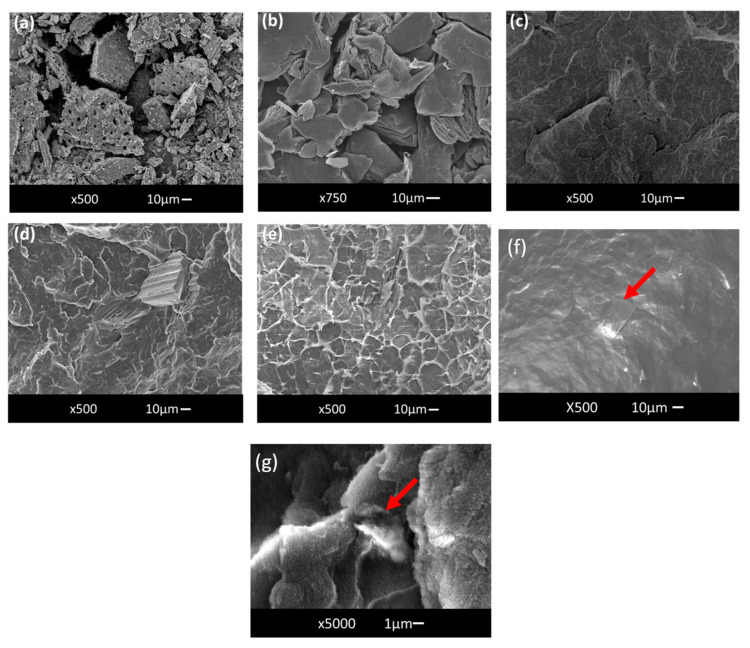
SEM Images of (**a**) Candlenut Derived Biochar; (**b**) Graphene; (**c**) Neat PP; (**d**) PP + 2 wt% BIOC; (**e**) PP + 0.5 wt% GN; (**f**) PP + 2% HEM (Red arrows pointing at HEM particle); (**g**) PP + 2% ROCH. (The red arrows indicate the location of the ROCH particles).

The far infrared emissivity of PP films incorporated with additives at 0.5 and 2 wt% from 4–15 µm with respect to black body radiation is depicted in Figure 2. Samples of films with additives were studied by SEM with the implementation of a mapping function to confirm the presence of additives in the film and see a distribution of particles in the matrixes. Result of SEM studies is presented in Figure 3. Overall, all samples with incorporated additives exhibited higher emissivity compared to the PP blank film. Using ANCOVA, since *p* < 0.001, the variation of emissivity results is significant (See Figure S5). Notably, the PP film with 2 wt% ROCH achieved the highest emissivity of 0.97. ROCH, primarily composed of iron oxide, exhibits various phonon modes related to Fe and O atomic vibrations. Thermal energy excites these phonons, leading to far infrared radiation emissions [41]. Conversely, the film with the lowest measured emissivity after adding additives was 0.87 for 0.5 wt% HEM. Interestingly, PP films with 0.5 wt% GN achieved an emissivity of 0.96, consistent with findings by Hu et al. [42]., who demonstrated that a small amount of graphene coating on polyurethane can significantly enhance the far infrared emissivity of the resulting composite. The high emissivity of graphene may be attributed to the presence of π electron optical transitions, enhancing absorption and emission in the infrared region [10].

When the concentration of additives increased from 0.5 to 2 wt%, the emissivity of the films also increased, ranging from +0.8% for PP films with BIOC to +8% for PP films with ROCH. Despite exhibiting lower emissivity than PP films with GN, ROCH, and HEM, PP films with BIOC demonstrated a notable increase in emissivity of up to +10.8% at 2 wt%. This enhancement could be attributed to the porous structure of biochar (as observed in Figure 3a), as higher porosity provides a larger surface area for the adsorption and reflection of electromagnetic radiation, resulting in higher emissivity [43].

A material with higher emissivity can efficiently convert heat energy from sunlight or the human body into electromagnetic radiation. A perfect emitter of heat, known as a black body, has an emissivity of 1. Thus, despite having lower emissivity than other additives, PP with 2 wt% BIOC achieved an emissivity of 0.90 with respect to black body radiation, higher than the far infrared emissivity of ceramic-embedded polymer previously reported [44]. An emissivity of 0.9 indicates that PP with 2 wt% BIOC can emit 90% of the blackbody radiation within 4–15 µm, compared to pristine PP, which emits only 81% of the black body radiation. Increasing the concentration of BIOC from 0.5 wt% to 2 wt% only marginally increased the emissivity by 0.01, suggesting a saturation limit in the effect of emissivity with the concentration of BIOC. This could be due to the relatively higher tendency of biochar to agglomerate at 2 wt% compared to 0.5 wt%.

### 2.3. Thermal Effusivity and Heat Retention Properties of PP Films with Functional Additives

**Figure 4:**
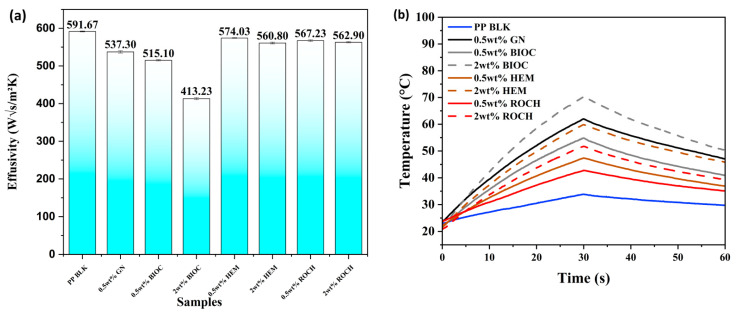
Thermal Properties of PP films with additives: (**a**) Thermal effusivity of PP films with 0.5 wt% and 2 wt% additives at 10 s touch time; (**b**) Heat retention of PP films with additives showing change of temperature difference from the initial temperature of films with 0.5 wt% and 2 wt% additives under infrared light for 30 s (0–30 s) and 30 s after infrared light off (30–60 s).

Thermal effusivity is defined as the capability of a material to transfer heat energy to its surroundings and is directly proportional to the thermal conductivity of the material [45]. The thermal conductivity of HEM, ROCH, and GN is higher than PP, thus an increase in thermal effusivity is expected after their incorporation into PP, as shown in Figure 4a. However, all samples incorporated with the functional additives generally exhibit lower thermal effusivity than pristine PP. Since *p* < 0.001, the null hypothesis is rejected, indicating that the variance in effusivity is statistically significant (See Figure S6). As the concentration of additives increases from 0.5 wt% to 2 wt%, the thermal effusivity decreases further. The reduction in thermal effusivity for samples incorporated with HEM, ROCH, and GN may be caused by poor interaction at the interface between the additives and PP (as seen in Figure 3e–g), which inhibits heat transfer between the composites, leading to lower thermal effusivity [46,47]. The poor filler/matrix interface when GN is added is illustrated in Figure 3e.

The lowest thermal effusivity is observed in PP films with 2 wt% BIOC, with a value of 413.23 W√s/m^2^K at 10 s. This low thermal effusivity from PP film incorporated with BIOC is attributed to the high porosity of biochar, as shown in Figure 3d. Bordoloi et al. [48] reported that the heat transfer of material was hindered after biochar was added into an organic phase change material due to the high porosity of biochar, which may introduce heat-resistant air voids. A material with higher thermal effusivity provides the wearer with a colder sensation, while a material with lower thermal effusivity gives the wearer a warmer feel. These results suggest that BIOC can potentially be used as an additive to textiles, providing a warming effect.

A lamp test was conducted following a previous study, where samples were exposed to an IR lamp and then cooled to observe the heat retention capabilities of the composites [49]. However, the testing time was adjusted to 30 s as the polymer samples were thinner compared to the fabrics reported in the previous study. Figure 4b illustrates the temperature difference of the PP films after exposure to the IR lamp for 30 s and subsequent cooling for 30 s after the IR lamp was switched off. The results indicated that all PP films with additives at both 0.5 and 2 wt% exhibited better heat retention capabilities than pristine PP. With an increase in the concentration of additives, the temperature increment upon exposure to the IR lamp was also enhanced. Films with 2 wt% BIOC achieved the highest temperature increase of +36.45 °C. Both films with HEM and ROCH had a lower temperature increment compared to the film with the carbon-based additives, with an increase of +25.97 °C and +17.84 °C at 2 wt%, respectively. Even after 30 s when the IR lamp was switched off, all films with additives displayed a higher net increase in temperature ranging from +11.39 °C to +28.86 °C compared to pristine PP, which showed a net increase of only +6.89 °C.

The rationale behind the higher temperature increment after IR lamp exposure may be attributed to the higher capability of GN and BIOC to absorb far infrared radiation. A study by Mak et al. [50] has demonstrated that graphene exhibits good far infrared absorption due to intraband transitions at low phonon energies. The high temperature increase of BIOC could be attributed to its low effusivity resulting from its porous structure. A material with low effusivity also has low thermal conductivity, meaning that heat will not be lost through the surroundings, thus accelerating the temperature increase when exposed to a radiation source [51].

### 2.4. Thermal Properties by DSC Analysis

**Figure 5:**
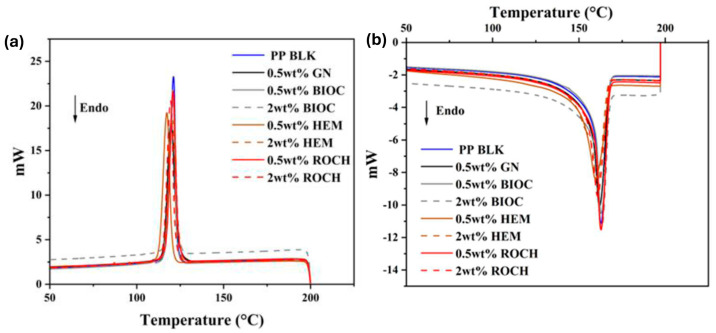
DSC Thermogram of PP films with additives: (**a**) Cooling thermogram from 200 °C to 30 °C; (**b**) Second heating thermogram from 30 °C to 200 °C.

The thermogram of the DSC analysis is depicted in Figure 5. The melting endotherm of the PP samples with additives is summarized in Table 1. Generally, the addition of additives up to 2 wt% did not alter the melting temperature compared to pristine PP, suggesting that the melting behavior of the composites resembles typical PP behavior. However, the addition of GN, BIOC, HEM, and ROCH resulted in increased melting energy and degree of crystallinity of PP. This rise in melting energy upon the addition of these additives is attributed to the incorporation of thermally stable additives into the PP matrix [52]. Moreover, the increase in crystallinity upon the addition of these additives implies that they facilitate the formation of spherulites and enhance the crystallization process [53]. The enhancement in crystallinity becomes more pronounced with increasing concentration of additives. The PP with 2 wt% HEM exhibited the highest melting energy and degree of crystallinity, while the PP with 0.5 wt% had the lowest. Although various studies have reported that the addition of these additives does not affect crystallization temperature, the findings contradict this notion. Instead, the results from this study indicate that the addition of additives, except for PP with 0.5 wt% HEM, had no effect on the crystallization temperature. This discrepancy may be attributed to the higher concentrations of additives used in previous studies, typically at least 10 wt%, whereas the lower concentrations used in this study may not be sufficient to facilitate enhanced crystallization.

Interestingly, the crystallization temperature (Tc) of 0.5 wt% HEM was lower than that of the other additives. However, when the concentration of HEM was increased to 2 wt%, the Tc returned to the level of pristine PP. Doumeng et al. [54] reported fluctuations in Tc with increasing concentrations of additives, suggesting that the decrease in Tc is caused by the additives hindering crystallite growth. Thus, at a concentration of 0.5 wt%, the decrease in Tc is attributed to HEM hindering the growth of crystallites, leading to the formation of imperfect crystals.

### 2.5. UV and NIR Shielding Properties of PP Films with Functional Additives

**Figure 6:**
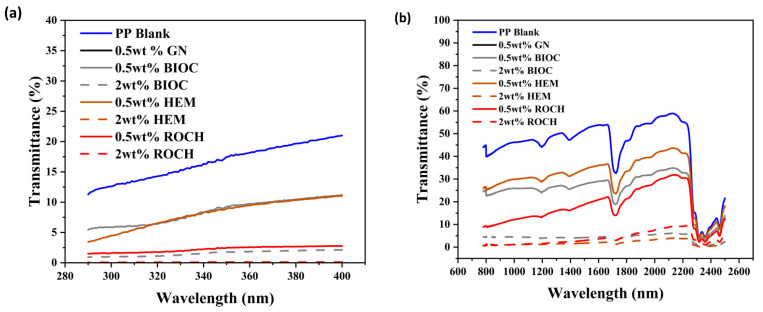
UV-Vis NIR characterization of PP films with additives: (**a**) UV spectra of radiation from 290–400 nm; (**b**) NIR spectra of radiation from 780–2500 nm.

UV-VIS NIR spectra as shown in Figure 6 were obtained to assess the UV and NIR protection properties of the PP films with additives. The percentage transmittance was used to calculate the UPF. The arithmetic mean of UVA, UVB, UVR, and NIR shielding is tabulated in Table 2. High NIR shielding percentages are important for preventing excessive heat buildup, particularly in applications like textiles, windows, or coatings. Effective NIR shielding can improve thermal comfort while reducing indirect skin damage caused by heat. The pristine PP showed no UV protection with a UPF of only 7.31 and UVR_AV of only 16.58%. When only 0.5 wt% of GN was added to PP, the film was able to achieve excellent UV and NIR protection with a UPF value of 58.05, UVR_AV of 2.22%, and NIR shielding of 82.58%. Lijun Qu reported that the enhanced UV protection after incorporation of graphene is due to the unique 2D planar structure of graphene which absorbs UV at shorter wavelengths and reflects UV at longer wavelengths [55]. The enhancement of NIR shielding after the incorporation of GN into the polymer matrix was due to the molecular dissipation of incident energy caused by color and the presence of numerous sp2-hybridized free electrons [56]. BIOC is also a suitable additive to be added to PP to enhance the UV and NIR protection properties. At a 0.5 wt% filler concentration, it can achieve a UPF of 15.79, a UVR_AV of 8.42% (which is considered good UV protection), and NIR shielding of 75.09%. Although still having a lower UPF value than pp films with HEM and ROCH, the protection capabilities of PP with BIOC films were enhanced when the filler concentration was increased to 2 wt%, which has a UPF value of 91.41 (UPF 50+, Excellent UV Protection), a UVR_AV of 1.56%, and NIR shielding of 95.85%. The absorption of UV/NIR light by carbonaceous material is dependent on the island size of the sp2 clusters. A larger cluster size can decrease the optical bandgap of the UV/NIR absorption which increases electromagnetic radiation absorption [57]. Marrot et al. [39] reported that the presence of defective graphene sp2 clusters is observed when carbonaceous waste is pyrolyzed with a minimum temperature of 600 °C. Exposure to UV can cause adverse human health effects such as sunburn, aging of the skin, and cancer [58]. The UPF achieved by adding 2 wt% BIOC is higher than previously reported UPF of zinc oxide-based sunscreens [59,60]. Long-term exposure to NIR rays can cause photoaging [61] and cataracts [62]. NIR shielding capabilities of various sunscreens that have claimed to have protection against NIR have not been reported for comparison. A significant decrease in the UVA_AV and UVB_AV percentages can help protect against sunburn and reduce the risk of skin cancer, respectively [12].

### 2.6. Mechanical Properties of PP Films with Functional Additives

The mechanical properties of pristine PP films and films with additives are shown in Table 3.

The tensile stress and elongation at break of the pristine PP film were 30 MPa and 4.22%, respectively, which aligns with the results reported by H. Bhunia [63]. Generally, all PP samples with additives exhibited a higher Young’s Modulus. For tensile strength, compared with pristine PP film, the addition of GN, HEM, and ROCH significantly decreased the ultimate tensile stress (UTS) and elongation at break of the PP film. This reduction in UTS and elongation at break may stem from stress concentration due to weak bonding between the PP matrix and these iron oxide-based additives, as iron oxide possesses a much higher surface energy than PP [64]. Additionally, the incorporation of inorganic fillers can impede the movement of PP chains, limiting the deformation of PP and energy absorption [65]. GN is prone to aggregation. This can reduce the dispersion of graphene within a PP matrix, leading to poor mechanical properties in the composite.

**Figure 7:**
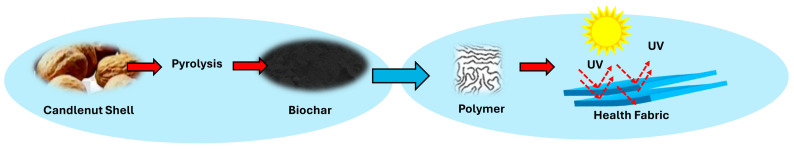
Schematic diagram of the potential application of biochar in polymer for health fabric.

Conversely, incorporating BIOC into PP, even when the concentration is increased from 0.5 wt% to 2 wt%, shows no change in UTS and only a slight reduction in elongation at break compared to pristine PP. GN exhibits a higher degree of graphitization and is expected to have better mechanical properties based on the results from Raman Spectroscopy. However, the presence of functional groups in the additives that can form a robust polymer-filler interface is a critical factor in achieving enhanced mechanical properties. The pyrolysis temperature of 600 °C in the presence of AC cannot completely eliminate polar functional groups such as C–O, C=O, COOH, and OH, which reduces the polymer-filler bonding interaction with a non-polar polymer such as polypropylene [66]. The poor polymer-filler interaction is the cause of the slight decrease in elongation at break compared to pristine PP, although it still remains more ductile when compared to other mineral-based additives in PP. Mechanical properties are crucial in textile materials to prevent permanent deformation under applied loads and repeated usage. The satisfactory performance of biochar in PP in terms of optical, mechanical, and effusivity properties indicates its potential for use in health fabric applications (Figure 7). By incorporating biochar in PP, fabrics can effectively block UV rays while also offering other beneficial properties like improved thermal regulation.

## 3. Materials and Methods

### 3.1. Preparation of Biochar and Functional Additives

To obtain biochar derived from candlenut shells (BIOC), the shells were first dried and then pyrolyzed at 600 °C for 5 h. Previous studies have shown that biochar with fewer oxygen functional groups demonstrates better compatibility with non-polar PP when used as filler additives in PP composites [67,68]. To enhance the affinity of the obtained candlenut shell biochar with non-polar PP, the biochar was subsequently mixed with an appropriate amount of activated carbon (AC), which was purchased from Sigma Aldrich, USA. The candlenut shell biochar underwent pyrolysis again at 600 °C for 60 min in the presence of AC to reduce the number of oxygen functional groups. The high surface area and porosity of AC can act as an adsorbent catalyst during pyrolysis, facilitating the removal of oxygen functional groups by adsorbing oxygen-containing molecules or radicals released during the thermal decomposition of the biochar. This represents an alternative energy-saving approach to reduce functional groups in biochar without the need for high-temperature biomass pyrolysis. Finally, the biochar was separated from the AC, washed, dried in an oven, and sieved using a 150-wire mesh sieve.

Hematite (HEM) was purchased from Mikon Co. (Singapore) and milled into powder using a micro powder pulverizer (Tencan GJ-1, Changsha City, China), then sieved using a 10 µm sieve. Indian red ochre (ROCH) was purchased from Konstantas (https://konstantas.com/, accessed on 1 April 2023). 96% pure graphene (GN) was purchased from Sigma Aldrich, St. Louis, MO, USA.

### 3.2. Preparation of PP Composite Films

BIOC, HEM, and ROCH were mixed with PP (LyondellBasell, The Netherlands) at 0.5 wt% and 2 wt%, respectively, at 180 °C using a lab-scale twin-screw extruder (Wuhan Ruiming, Wuhan, China) under controlled rotational speed. The temperatures for the feed zone, compression zone, and melt zone were set to 120 °C, 170 °C, and 180 °C, respectively. The rationale behind these settings was to preheat the material in the feed zone, gradually melt it in the compression zone, and finally ensure that the plastic is homogeneously melted in the extruder. The extruded filaments were air-dried and pelletized. The pellets were then hot-pressed at 180 °C for 10 min using a 150 × 150 × 0.5 mm square mold. A blank sample (PP BLK) with sample amount of PP alone and a sample with 0.5 wt% GN was also extruded using the same conditions and molded using hot press as a control for comparison. The use of only 0.5 wt% graphene is due to its high cost, making higher concentrations uneconomical, as well as the tendency for agglomeration at higher concentrations. 

### 3.3. UV and NIR Protection Properties

The UV radiation spectra from 290–400 nm and the NIR radiation spectra from 780–2500 nm of the blank PP films and PP films with additives were obtained using a UV-VIS NIR spectrometer (Agilent Cary 60 UV–Vis spectrophotometer, Cary, NC, USA) and taking the average of 3 samples. The UV protection properties were analyzed using a method from the AS/NZS 4399:1 996 standard [69]. The arithmetic means of the UVA transmittance (UVA_AV_), UVB transmittance (UVB_AV_), UV radiation (UVR_AV_) and ultraviolet protection factor (UPF) were calculated according to the following formulas:

(1) $${\mathrm{UVA}}_{\mathrm{AV}}\;(\%)=\frac{T_{315}+T_{320}\ldots \ldots ..T_{395}+T_{400}}{18}$$

(2) $${\mathrm{UVB}}_{\mathrm{AV}}\;(\%)=\frac{T_{290}+T_{295}\ldots \ldots ..T_{310}+T_{315}}{6}$$

(3) $${\mathrm{UVR}}_{\mathrm{AV}}\;(\%)=\frac{T_{290}+T_{295}\ldots \ldots ..T_{395}+T_{400}}{23}$$

(4) $$\mathrm{UPF}=\frac{\sum_{290}^{400}E(\lambda )\times S(\lambda )\times \Delta \lambda }{\sum_{290}^{400}E(\lambda )\times S(\lambda )\times T(\lambda )\times \Delta \lambda }$$

where *T(λ)* is the percentage spectral transmittance of the sample at wavelength λ, *E(λ)* is the relative erythemal spectral effectiveness, *S(λ)* is solar spectra irradiance in W m^−2^·nm^−1^, and Δ*λ* is the measured wavelength interval in nm. The UPF protection categories are adopted from the AS/NZS 4399:1 996 standard.

The NIR shielding of blank PP films with additives was calculated by comparing the area under the curve (AUC) of the transmittance spectra with 100% transmittance and calculated using the following formula:

(5) $$\mathrm{NIR}\;\mathrm{shielding}\;(\%)=(1-\;\frac{AUC\;of\;samples\;at\;780\text{–}2500\;nm}{AUC\;of\;spectra\;with\;100\%\;transmittance\;at\;780\text{–}2500\;nm})$$

### 3.4. Thermal Analysis and Heat Storage

The thermal properties of the PP composites were analyzed using the Q10 DSC (TA Instruments, New Castle, DE, USA) using a heat–cool–heat method under the ISO 11357-1:2023 standard [70] and average curve of 3 samples was reported. Briefly, approximately 5 mg film samples were heated from 30 to 200 °C at a rate of 10 °C/min, and subsequently cooled to 30 °C at the same cooling rate of 10 °C/min. The samples were re-heated using the same conditions as those employed during the first heating cycle. Melting temperature (*Tm*), crystallization temperature (*Tc*), and heat of fusion (Δ*H_m_*) of the samples were then obtained from the second heating process and analyzed. The degree of crystallinity (*X_c_*) was calculated using the following formula:

(6) $$X_{C}=\frac{∆H_{m}}{∆H_{m100}(1-w_{f})}$$

where Δ*H_m_* is the heat of fusion of the sample; Δ*H_m100_* is the heat of fusion of 100% PP polymer, which is 204 J/g [33]; and *W_f_* is the weight fraction of additives.

**Figure 8:**
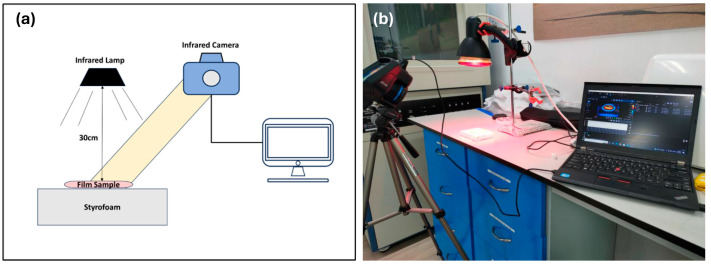
(**a**) Schematic diagram and (**b**) actual setup images illustrating the experimental arrangement used to study the heat capacity of polypropylene films with mineral powders.

A heat storage test was conducted in accordance with a previous study, where the samples were exposed to an IR lamp and then allowed to cool to evaluate the heat retention capabilities of the composites [49]. An infrared lamp test (refer to Figure 8) was used to determine the heat storage and release of the PP films with additives. The power of the infrared (IR) lamp was 150 watts with a wavelength from 0.75 to 5 µm and a peak wavelength of 4 µm. The samples were tested with exposure to IR for 60 s and then another 60 s after removal from exposure to IR. The distance between the IR lamp and the sample was 30 cm. The IR camera FLIR E86 (Teledyne FLIR, Wilsonville, OR, USA) was used to record the temperature of samples.

### 3.5. Other Characterisations

Raman spectroscopy was done with a WITEC Confocal Raman (Oxford instruments, Oxford, UK) with a laser wavelength of 488 nm. A 20× objective lens was used and the scanning done from −2000 cm−1 to 3000 cm−1. The cross-sections of PP composite films were obtained after being cryo-fractured with liquid nitrogen. The fractured surface of the PP composites were coated with gold using a sputter coater and observed under a field emission scanning electron microscopy (FE-SEM) with a voltage of 5 kV (JEOL 7600F, Japan). Fourier Transform Infrared Spectroscopy was performed on GN and BIOC. Samples of powder were pelletized with KBr and analyzed using a FTIR spectrometer (Perkin Elmer Frontier, USA) with a resolution of 4 cm^−1^ and 16 scans through a slide holder with a 1 cm diameter hole. Emissivity of the films in the range of 4–15 µm wavelength was measured at 34 °C using a Far Infrared Emissivity Analysis System (HOTECH EMS302M, Taiwan).

The measurement of the emissivity of films was relative to 1, which is the emissivity of a perfect black body, which emits all the thermal radiation it can at a given temperature, while 0 represents a perfect reflector, which emits no thermal radiation. Thermal effusivity of the PP films was obtained at 2 s and 10 s at a temperature of 24 °C with a thermal effusivity meter (Thermtest, Hanwell, NB, Canada).

ANCOVA was performed to analyze the emissivity results and ANOVA was used for the effusivity results; both were conducted using DATAtab statistical online calculator (https://datatab.net/, accessed on 14 September 2024). In the ANCOVA analysis, emissivity was the dependent variable, with the sample serving as the covariate. For the ANOVA analysis of effusivity, effusivity was treated as the metric variable, while the sample was the nominal variable.

A tensile test was performed using MTS Criterion Model 43 (Criterion, New York, NY, USA). Five strips of film were tested according to ASTM D882 standard [71] with a width of 1.5 cm and grip separation of 10 cm. The testing speed used was 12.5 mm/min.

## 4. Conclusions

In this study, candlenut-derived biochar, hematite, and Indian red ochre were incorporated into polypropylene (PP) at 0.5 and 2 wt% and their performance was compared against 0.5 wt% graphene/pp composite. The addition of all additives enhanced the FIR emissivity in the range of 5–14 µm, with the highest value recorded as 0.97 for the composite with red ochre. Additionally, all additives in PP films at 2 wt% demonstrated excellent UV protection (UPF +50), with the highest protective coefficient achieved by red ochre. Furthermore, all composites provided good NIR protection.

Moreover, all additives increased the heat accumulation capacity of the PP film when exposed to a FIR irradiation source. PP with BIOC exhibited superior heat capacity, with these films achieving a temperature increase of +48.89 °C at 2 wt% when exposed to a FIR source for 30 s, suggesting a 3.3-fold higher increase than pristine PP. The potential of PP films with BIOC to be used for warm clothing material is further supported by having the lowest thermal effusivity at a concentration of 2 wt%, suggesting it can provide a warmer feel than the other additives.

Furthermore, PP films with BIOC exhibited better thermal and mechanical properties than the other additives, as adding BIOC did not significantly change the mechanical properties of PP film. Despite these promising results indicating that BIOC is a potentially suitable additive for FIR textile applications, further studies need to be conducted on fabric incorporated with these additives. The fabrication process of yarn and polymer film differs, and it may influence the properties investigated in this study.

## Figures and Tables

**Table 1:** DSC melting temperatures (Tm), crystallization temperature (Tc), and degree of crystallinity (*X_c_*) with standard deviations of PP films with 0.5 and 2 wt% additives.

Films	Tm (°C)	Tc (°C)	Δ*H_m_* (J/g)	*X_c_* (%)
PP BLK	162.79 ± 0.01	120.85 ± 0.25	77.88 ± 0.33	37.26 ± 0.16
0.5 wt% GN	161.76 ± 0.17	119.77 ± 0.02	88.89 ± 1.30	44.77 ± 0.65
0.5 wt% BIOC	162.92 ± 0.11	120.79 ± 0.09	87.51 ± 0.48	42.08 ± 0.23
2 wt BIOC	163.29 ± 0.16	120.87 ± 0.18	88.88 ± 0.58	43.39 ± 0.29
0.5 wt% HEM	159.69 ± 0.08	117.64 ± 0.35	86.58 ± 1.92	41.63 ± 0.92
2 wt% HEM	160.87 ± 0.30	120.46 ± 0.23	95.25 ± 0.75	46.50 ± 0.37
0.5 wt% ROCH	160.48 ± 1.54	121.12 ± 0.15	76.14 ± 0.85	36.61 ± 0.41
2 wt% ROCH	160.72 ± 0.11	119.36 ± 0.04	81.46 ± 2.02	39.77 ± 0.99

**Table 2:** Mean ultraviolet protection factor (UPF), UPF protection category, mean UVA, UVB, ultraviolet radiation (UVR) transmittance, and percentage of NIR shielding of the films with additives.

Films	UPF	UPF Protection Category	UVA AV, (%)	UVB AV, (%)	UVR AV, %	NIR Shielding, %
PP BLK	7.31	No protection	17.71	12.74	16.58	56.02
0.5 wt% GN	58.05	Excellent protection	2.40	1.61	2.22	82.58
0.5 wt% BIOC	15.79	Good protection	9.14	5.89	8.42	75.09
2 wt% BIOC	91.41	Excellent protection	1.72	1.00	1.56	95.85
0.5 wt% HEM	8.03	No protection	9.03	4.72	17.73	69.59
2 wt% HEM	1142.38	Excellent protection	0.11	0.08	0.11	98.19
0.5 wt% ROCH	58.05	Excellent protection	2.40	1.61	2.22	82.58
2 wt% ROCH	2529.36	Excellent protection	0.06	0.04	0.05	96.31

**Table 3:** Ultimate Tensile Strength, Elongation at Break, and Young’s Modulus with standard deviations of PP Films with additives.

Films	Ultimate Tensile Strength, (MPa)	Elongation at Break, (%)	Young’s Modulus (GPa)
PP BLK	29.36 ± 0.47	4.20 ± 0.08	1.71 ± 0.07
0.5 wt% GN	18.20 ± 1.13	1.37 ± 0.19	1.83 ± 0.16
0.5 wt% BIOC	29.94 ± 0.83	3.70 ± 0.42	1.87 ± 0.04
2 wt% BIOC	31.45 ± 0.50	3.47 ± 0.29	2.20 ± 0.10
0.5 wt% HEM	16.24 ± 2.88	1.40 ± 0.00	1.78 ± 0.09
2 wt% HEM	11.91 ± 1.60	0.73 ± 0.21	2.12 ± 0.05
0.5 wt% ROCH	8.00 ± 3.30	0.40 ± 0.08	1.91 ± 0.21
2 wt% ROCH	6.00 ± 2.62	0.40 ± 0.16	1.85 ± 0.09

## Data Availability

The authors declare that the data supporting the findings of this study are available within the paper and its Supplementary Information files. Should any raw data files be needed in another format they are available from the corresponding author upon reasonable request.

## Supplementary Materials

The following supporting information can be downloaded at: https://www.mdpi.com/article/10.3390/molecules29204798/s1, Supplementary Figure S1: High-resolution optical microscope images for (a) Neat PP (b) PP + 0.5 wt% graphene; (c) PP + 2 wt% candlenut derived biochar; (d) PP + 2 wt% hematite; (e) PP + 2 wt% Indian Red Ochre. Supplementary Figure S2: Particle size of Hematite and Indian Red Ochre. Supplementary Figure S3: Fe Ka1 mapping for PP + 2%HEM. Supplementary Figure S4: Fe Ka1 mapping for PP + 2%ROCH. Supplementary Figure S5: ANCOVA statistical analysis of emissivity results using DATAtab Statistics Software. Supplementary Figure S6: ANOVA statistical analysis of thermal effusivity results using DATAtab Statistics Software.
