# Supplementary material for: Investigation of Far Infrared Emission and UV Protection Properties of Polypropylene Composites Embedded with Candlenut-Derived Biochar for Health Textiles
Source: Molecules. 2024 Oct 10;29(20):4798. doi: 10.3390/molecules29204798 (PMC11509977; doi:10.3390/molecules29204798)
Supplement: Supplementary file 1 [file molecules-29-04798-s001.zip › molecules-3139393-supplementary.pdf]

Investigation of Far Infrared Emission and UV Protection Properties of Polypropylene Composites Embedded with Candlenut-Derived Biochar for Health Textiles

Rayland Jun Yan Low [1], Pengfei He [1], Junianto [1], Ningyu Qiu [1], Amanda Jiamin Ong [1], Hong Han Choo [1], Yosia Gopas Oetama Manik [2], Rikson Siburian [2], Ronn Goei [1], Stephen Francis Burns [3], Alfred Iing Yoong Tok [1], Vitali Lipik [1*] and Boon Peng Chang [1*]

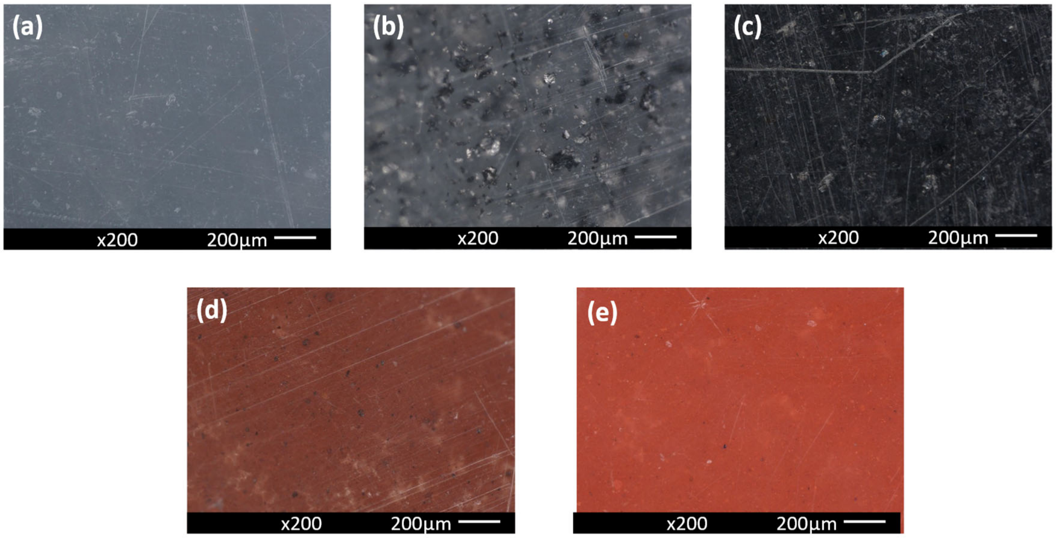

Supplementary Figure S1: High resolution optical microscope images for (a) Neat PP (b) PP+0.5wt% graphene (c) PP+2wt% candlenut derived biochar (d) PP+2wt% hematite (e) PP+2wt% Indian Red Ochre

Supplementary Figure S2: Particle size of Hematite and Indian Red Ochre

Minerals	d_{10} (µm)	d_{50} (µm)	d_{90} (µm)
HEM	0.52	2.50	5.39
ROCH	0.20	2.60	6.72

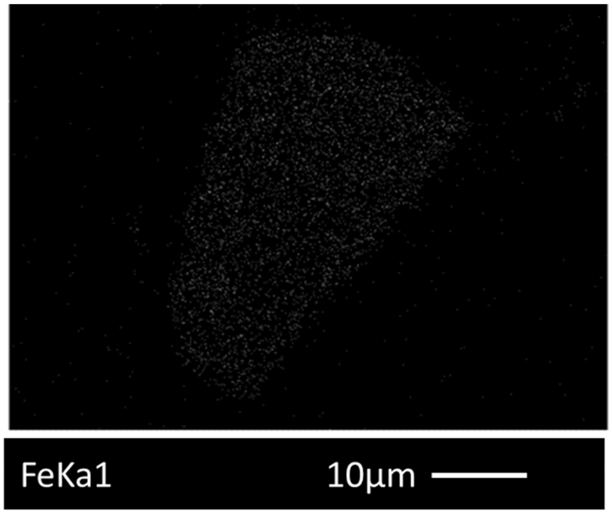

Supplementary Figure S3: Fe Ka1 mapping for PP+2%HEM.

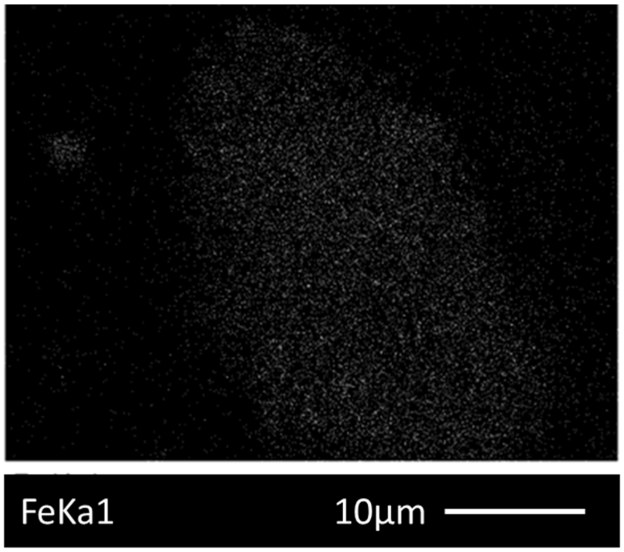

Supplementary Figure S4: Fe Ka1 mapping for PP+2%ROCH.

ANCOVA

	Sum of Squares	df	Mean Square	F	p
Samples	0.02	6	0	6.72552944538478e+27	<.001
Error	0	7	0		

Supplementary Figure S5: ANCOVA statistical analysis of emissivity results using DATAtab Statistics Software.

ANOVA

	Sum of Squares	df	Mean Square	F	p
Sample	66668.58	7	9524.08	1272.36	<.001
Residual	119.77	16	7.49		
Total	66788.35	23			

Supplementary Figure S6: ANOVA statistical analysis of thermal effusivity results using DATAtab Statistics Software.
